# Punch Edge Topological Design for Reduction of Work Hardening Damage in Shearing of Non-Oriented Electrical Steel Sheets

**DOI:** 10.3390/ma18040878

**Published:** 2025-02-17

**Authors:** Ryoma Okada, Kentaro Ito, Tatsuya Funazuka, Tatsuhiko Aizawa, Tomomi Shiratori

**Affiliations:** 1 Graduate School of Science and Engineering, University of Toyama, Toyama 930-8555, Japan; m23c1422@ems.u-toyama.ac.jp (R.O.); m23c1411@ems.u-toyama.ac.jp (K.I.); 2 Academic Assembly Faculty of Engineering, University of Toyama, Toyama 930-8555, Japan; funazuka@eng.u-toyama.ac.jp; 3 Surface Engineering Design Laboratory, Shibaura Institute of Technology, Tokyo 144-0045, Japan; taizawa@sic.shibaura-it.ac.jp

**Keywords:** shearing, punching, non-oriented electrical steel

## Abstract

A new shearing tool is necessary to reduce the iron loss of motor cores by minimizing the work hardening damage on the sheared non-oriented electrical steel sheets. The punch edge topology and the clearance between the punch and the die were controlled to investigate their influence on the sheared surface condition and the work hardening damage of steel sheets. A non-oriented electrical steel sheet with the thickness of 500 µm was used and sheared at the speed of 5 mm/s. After that, the sheared surface was investigated. In particular, hardness mapping was utilized to quantitatively analyze the work-hardened area of the sheared steel sheets and the dissipation of the plastic work. Among the four punch edge topological configurations explored, the nano-grooved punch employed straight along the shearing direction reduced the damage dealt to the sheared steel sheets and the plastic dissipation work to one-third compared to conventional punches.

## 1. Introduction

In the pursuit of SDGs with carbon neutrality, electric vehicles with highly efficient motors are in high demand to reduce energy consumption [1]. The motor cores are made from non-oriented electrical steels with magnetic isotropy. The energy dissipation by the loss of iron induced in them has become one of the most essential issues in related engineering processes. This loss is mainly attributed to the shearing-induced work hardening damage deal to the electrical steel sheets [2,3,4]. The plastic strains and residual stresses caused by the work hardening process pose a significant risk of worsening the magnetic domain structure. This deterioration induces hysteresis loss and eddy current loss [5]. In particular, the hysteresis loss caused by plastic straining results in magnetic anisotropy. Magnetization processes, characteristic of the damaged zone width, are influenced by the value of the applied magnetic field strength. A link can be established between the damaged zone’s magnetic and mechanical properties because an increased hardness value and bad magnetic properties usually characterize the areas most affected by cutting [6]. Hence, the shearing process must be improved to minimize the affected zone on the non-oriented electrical steel sheets in the shearing process and to reduce the iron loss.

In the shearing tool design, the clearance between the punch and the die plays a role in the shearing performance [7,8]. A larger clearance induces a bending moment applied to the work material, enhances work hardening, and deteriorates its magnetic properties. On the other hand, a smaller clearance is preferable in reducing work hardening, causing little degradation of magnetic properties [9,10]. However, shearing in low clearance often induces a secondary fracture surface and shortens the tool life. The punch edge configuration influences the shearing process. As stated in [11,12], a mechanically ground edge yields a burnished surface in shearing; the sheared surface quality deteriorates with its abrasive wearing. The work hardening area is exaggerated to be three times larger than the work sheet thickness when using a mechanically ground punch edge [2]. When using a carbide punch with an edge sharpened via argon-ion beam polishing, the work hardening area is much reduced in the shearing of AISI304 steel sheets [13,14]. As reported recently in [15,16,17,18], carbide and nitrided punches with periodically nano-grooved edges via short-pulse laser texturing improved the sheared surface quality by reducing the abrasive and adhesive wear of the punch. However, there has been no quantitative evaluation of the optimization of the clearance and punch edge conditions in the shearing of a non-oriented electrical steel sheet.

The present paper aims to quantitatively analyze the effect of the clearance and tool edge conditions on the minimization of work hardening in the shearing of non-oriented electrical steel sheets to identify a shearing condition that will significantly reduce iron loss. Four types of shearing punches are employed to analyze, experimentally, the effect of the clearance and punch-edge topological configurations on the work hardening damage incurred in the shearing of the non-oriented steel sheets. Using the specified experimental procedure, a die set with three different clearances is utilized. Four punches are prepared such that each of the experiments has different edge configurations, namely, a mechanically ground edge, an ion-milled edge, and a periodically nano-grooved edge with two groove orientations (20° and 90°).

From the experiments, it was found that the nano-grooved punches minimize the work hardening damage to work sheets in the shearing process. In addition, the nano-grooved edge, either straight along the shearing direction or at an angle of 90° against its edge, minimized the plastic dissipation work through the localization of the stress concentration and through control of local shearing.

## 2. Methods and Materials

### 2.1. Shearing Process

**Figure 1:**
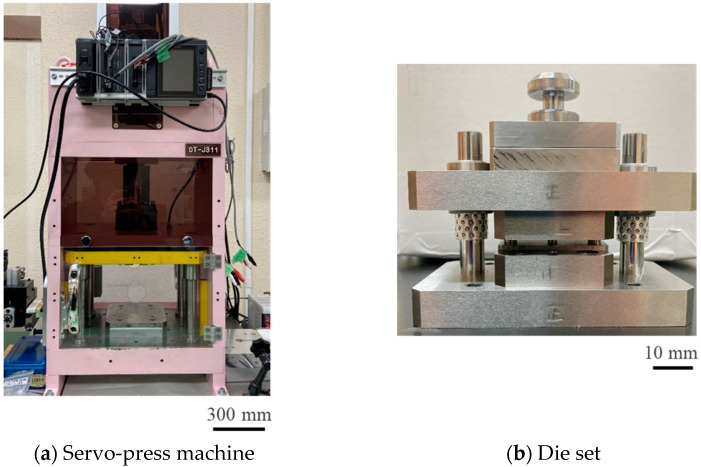
Experimental setup for the dry punching of non-oriented electrical steel sheets: (**a**) a CNC (computer numerical control) stamper; (**b**) a die set.

The screw servo stamping system (Precise Stamping Laboratory, DT-J311; Tokyo, Japan) was utilized to punch out the work materials, as shown in Figure 1a. The stroke was measured using the laser displacement meter (KEYENCE, SI-F01; Tokyo, Japan) and data acquisition system (KEYENCE, NR-600; Tokyo, Japan). Non-oriented Fe-3Si electrical steel sheets, with a thickness of 500 μm (NIPPON STEEL Corp., 50H310; Tokyo, Japan), were used as the work material. The WC (Co) die in Figure 1b and the work piece were inserted into a die holder. An SKD11 punch was inserted into the center hole of the punch guide and fastened to the die holder. The punch diameter was 5 mm. Three types of clearances were prepared to investigate the effect of clearance (δ) on work hardening; δ = 25 μm, i.e., 5% of work piece thickness (h), as a normal condition; δ = 5 μm, i.e., 1% of h; and δ = 2.5 μm.

The loading speed was constant at 5 mm/s. Two shearing experiments were conducted: short-shot at a 30% reduction in thickness and fully punching out.

### 2.2. Punch Edge Configurations

**Figure 2:**
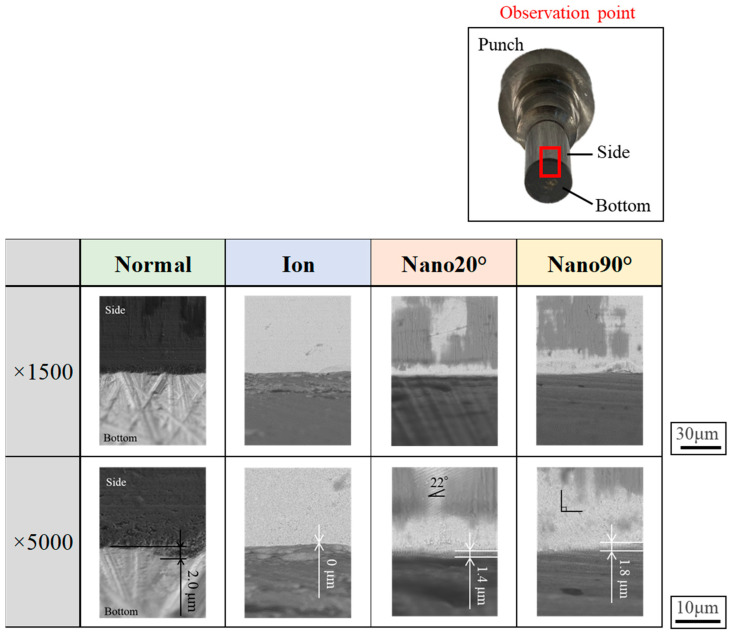
Four types of shearing punches with different edge configurations. Low magnification 1000x SEM images are listed in the upper row, and high magnification 5000x SEM images are listed in the lower row.

Four types of punches were used in this experiment, as listed in Figure 2: a normal punch with a mechanically ground edge; an ion-beam-treated punch with an edge sharpened by argon-ion irradiation; Nano20° and Nano90° punches with nano-grooved edges, with each width being 300 nm on their side surface at orientations of 20° and 90°, respectively, against their edges; and a femtosecond laser with a wave length of 515 nm, a pulse width of 200 fs, and a repetition rate of 20 MHz were utilized for nano-grooving [17]. 

### 2.3. Evaluation on Quality of Sheared Electrical Steel Sheets

The area near the right side of sheared hole was observed by SEM (JCM-7000, JEOL; Tokyo, Japan). The fractured surface area ratio was calculated using image processing software (mediBang Paint Pro ver. 2023.06, Photoshop ver. 26.3). The boundary of the fractured area was estimated via image processing. One pixel of the image was approximately 1 µm; the digitization error was 0.2%.

### 2.4. Mechanical Characterization

**Figure 3:**
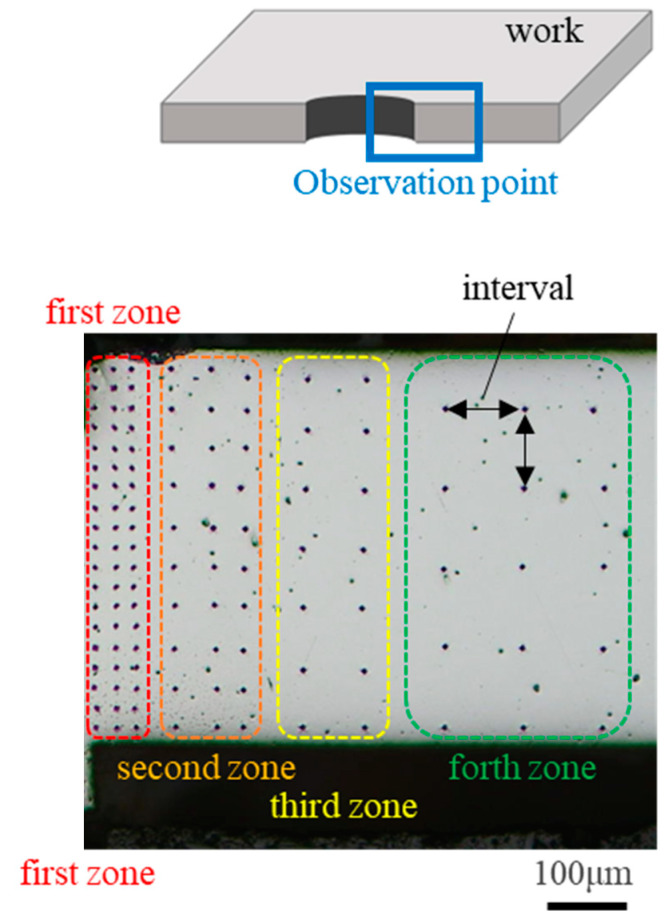
Hardness mapping to evaluate the affected zone of the sheared electrical steel sheets using four types of punches.

The work hardening zone of sheared electrical steel sheets was determined from the Vickers hardness map after cutting and polishing near the right side of the punched hole. The Vickers hardness tester (HM-100, Mitutoyo; Kawasaki, Japan) was used for mapping. The load was applied by 0.1 N for 10 s. The mapping scheme is shown in Figure 3. The hardness map was measured at three points with an interval of 25 μm on the first zone, at three points with an interval of 50 μm on the second zone, at two points with an interval of 75 μm on the third zone, and at three points with an interval of 100 μm on the forth zone. This hardness map is depicted by ten color-coded levels in each 25 HV. The personal error in measurement was suppressed to be lower than 15 HV, or 6% of the reference hardness of 250 HV for the electrical steel sheet without work hardening damages.

### 2.5. Estimate on the Plastic Work

To obtain material properties, a 1/2-scale JIS 13B tensile test specimen was used for tensile testing. The stress–strain curves were obtained at a strain rate of 0.1 mm/s.

The plastic work (W) induced in the sheared sheets is estimated using the measured hardness map. This W is defined by

(1) $$W=\int_{V}w\times dv$$

where w is the work density, dv is the representative volume of each measuring zone in the hardness mapping, and V is the total volume of the plastically deforming work materials, excluding the elastically deforming volume. The stress–strain curve of the non-oriented electrical steel sheet is modeled by the rigid plastic model; e.g., $\sigma =\sigma_{y}+K\times \varepsilon$, where σ is the stress, K is the work hardening modulus, $\sigma_{y}$ is the yield stress, and ε is the plastic strain. These K and $\sigma_{y}$ values are given by the uniaxial tensile test. Then, the plastic work density is represented by the following.

(2) $$w=\frac{1}{2}\;\left(\sigma_{y}+\sigma \right)\times \varepsilon =\frac{1}{2K}\times \left(\sigma +\sigma_{y}\right)\times \left(\sigma -\sigma_{y}\right)$$

**Figure 4:**
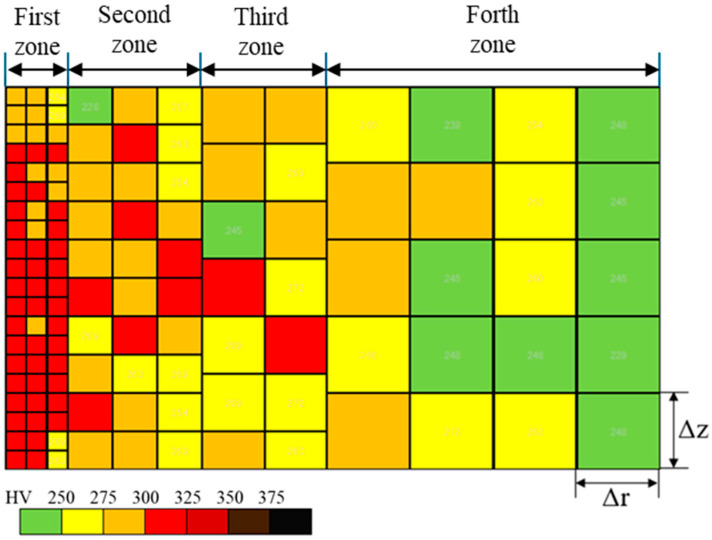
Discretization of the work hardening area induced in the right-hand side of the sheared steel sheet. The m-th square element has the m-th measuring point of hardness.

In the case of the hardness mapping in Figure 4, V was discretized into M representative elements, including each measuring point of hardness. Then, the plastic work is calculated by

(3) $$W=\sum_{m=1}^{M}\left(\frac{1}{2K}\;\right)\times \left({\sigma_{m}}^{2}-{\sigma_{y}}^{2}\right)\times 2\pi r_{m}\;\Delta r\Delta z$$

where r_m_ is a radius of m-th measuring point from the symmetric axis, and Δr and Δz are pitches in the radial and axial directions, respectively, in Figure 4.

## 3. Experimental Results

### 3.1. Sheared Surface Observation

**Figure 5:**
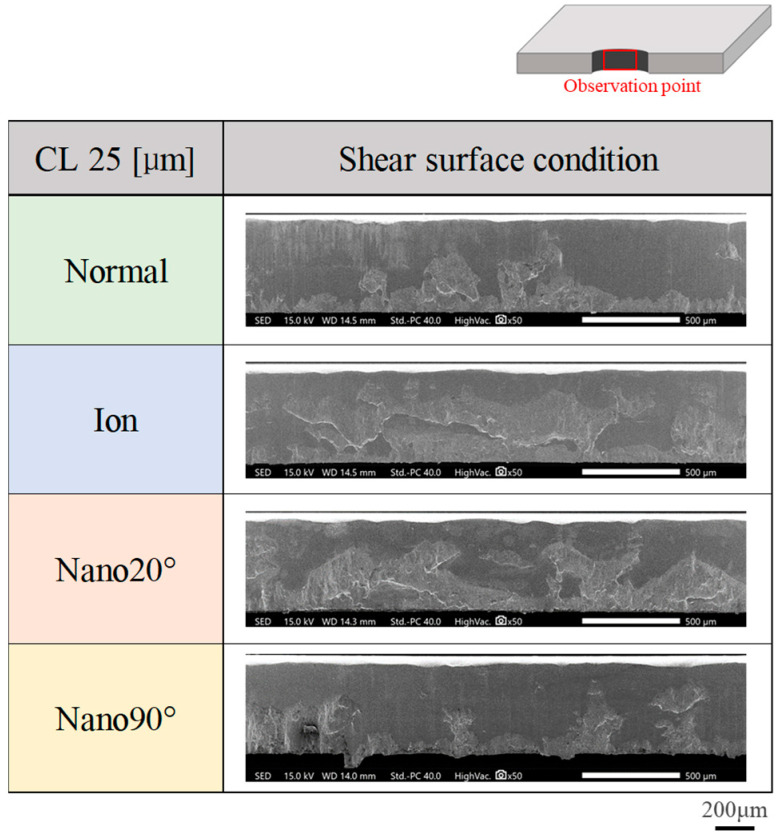
Comparison of sheared surface conditions among four punches at a clearance of CL25 μm.

**Figure 6:**
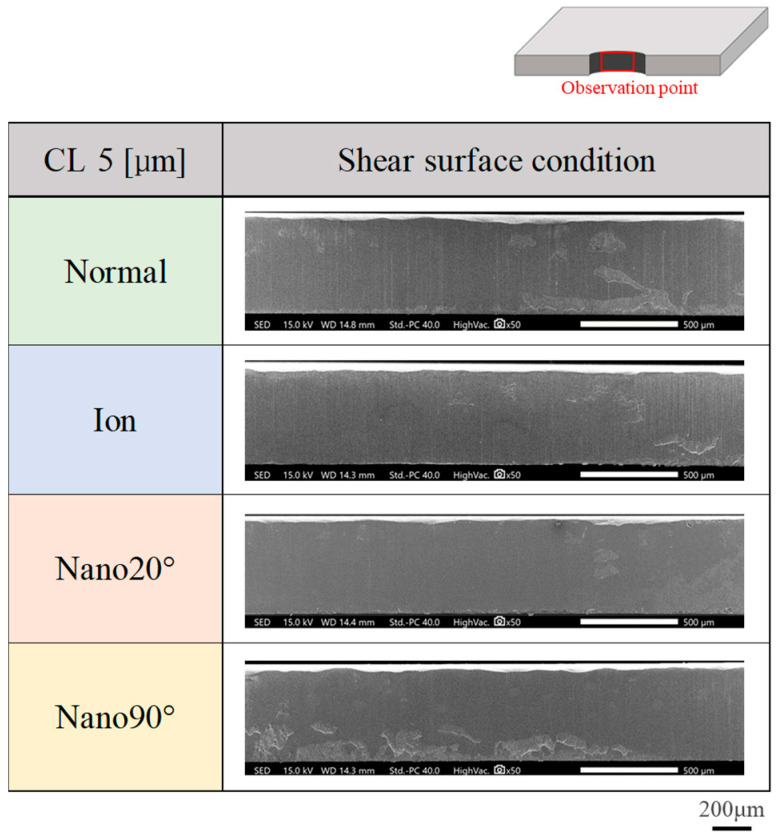
Comparison of sheared surface conditions among four punches at a clearance of CL5 μm.

**Figure 7:**
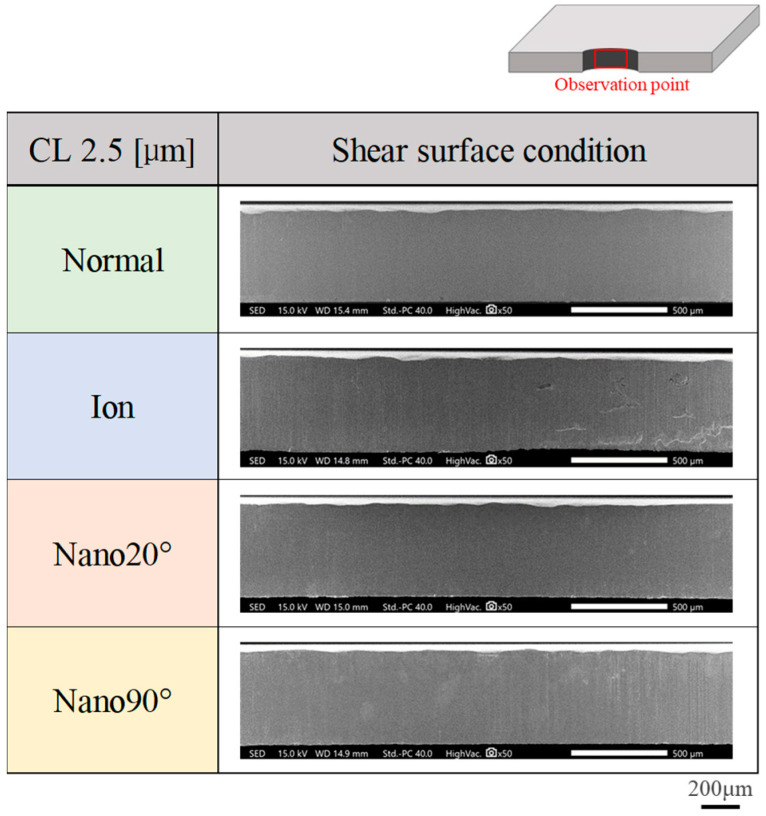
Comparison of sheared surface conditions among four punches at a clearance of CL2.5 μm.

The sheared surface conditions attained using four punches were evaluated via SEM observation. Figure 5 compares four sheared surface conditions where δ = 25 μm or at CL25 μm. The fracture surface extends heterogeneously. In the case of CL5 μm in Figure 6, the fractured surface area ratio decreases and the burnished surface area ratio increases. Less-fractured surface areas are detected at CL2.5 μm in Figure 7.

**Figure 8:**
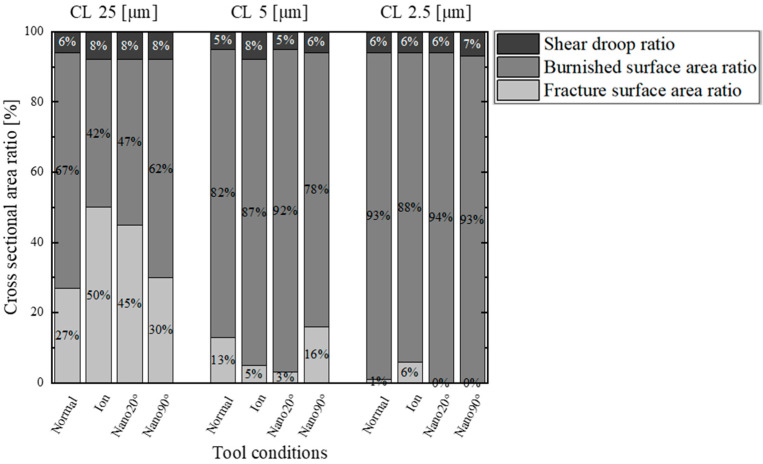
Comparison of the fractured and burnished surface area ratios with the shear droop ratio for the four punches at CL25 μm, CL5 μm, and CL2.5 μm.

The variation of these fractures and the burnished surface area ratios attained by changing the clearance are listed in Figure 8, and the four punches are compared together with the measured shear droop ratio. At CL25 μm, the fractured surface area ratio exceeds 27% for every condition. Reducing the clearance down to CL5 μm, the fractured surface area ratio significantly decreases to 3% in the case of Nano20°. At CL 2.5 μm, no fractured surface areas are detected when using the Nano20° and Nano90° punches.

### 3.2. Work Hardening Zones on the Sheared Surfaces

**Figure 9:**
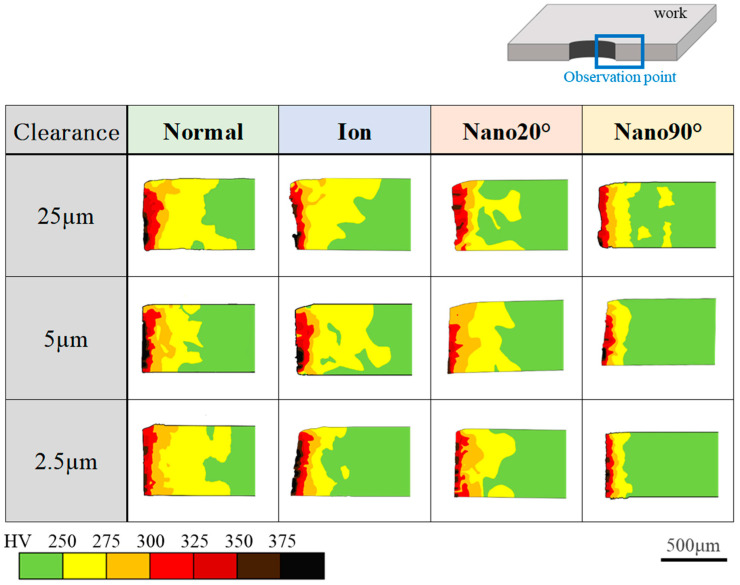
Hardness comparison of the work hardening zones for each clearance of CL25 μm, CL5 μm, and CL2.5 μm for the four punches.

**Figure 10:**
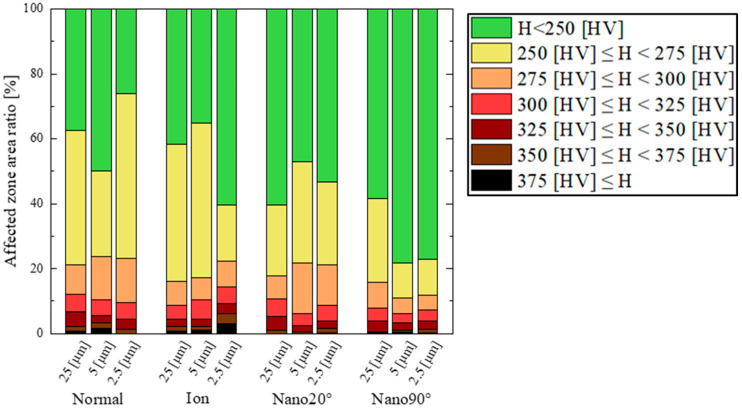
Hardness contribution of six work hardening and elastic zone ratios on the whole cross-sectional area at each clearance for the four punching conditions.

The work hardening zone is defined as the area with a hardness greater than 250 HV in the hardness map. Each zone is compared at each clearance for each punch condition in Figure 9. The elastic zone is represented by the green color. Based on the hardness map in Figure 9, Figure 10 shows the affected area ratio of six work hardening zones and an elastic zone.

When using a normal punch, a larger work hardening zone is detected irrespective of the clearance. It should be noted that since the red-colored zone with hardness greater than 300 HV is detected at the nearest to edge, work hardening concentrates in the vicinity of the punch edge.

In the case of the ion punch, the work hardening zone decreases with the reduction in clearance. The work hardening zone with a hardness greater than 375 HV is detected in the vicinity of sheared surface. It is enhanced with the reduction in clearance. This is because of the severe plastic flow around the sharpened punch edge.

When using the Nano20° punch, a large work hardening zone fluctuates by itself on the cross-section, irrespective of clearance. It should be noted that the highest hardened zone, i.e., the zone with a hardness greater than 375 HV, is only detected pointwise in the vicinity of the sheared surface.

In the case of the Nano90° punch, the work hardening area greater than 250 HV, shown in yellow color, is concentrated in the vicinity of the hole and is minimized when the clearance is lower than 5 μm.

As summarized in Figure 10, the fraction of each work hardening zone in the whole cross-sectional area is compared at each clearance and with each punching condition. When using the normal punch, the yellowed colored zone fraction becomes the maximum (51%) at CL2.5 μm. In case of the Nano90° punch, the yellowed colored zone fraction becomes the minimum (11%) at CL5 μm and CL2.5 μm. This fraction is less than the minimum fraction using the ion beam (i.e., 17% at CL2.5 μm) by 35%, and it is half of the minimum fraction attained using Nano20° (i.e., 22% at CL25 μm).

Table 1 lists the contribution of the elastic zone and the medium and high work hardening zones to the total cross-sectional area. When using the Nano90° punch, the cross sectional area is determined to be elastic; the medium work hardening zone is limited by 15%; and the high work hardening zone is reduced to less than 5%. Most of the errors come from the measured area at the first zone in Figure 3, with the pitch of 25 µm. The maximum error area was estimated to be 4%.

### 3.3. Measurement of Work Hardening Zone for Short-Shot Specimens

**Figure 11:**
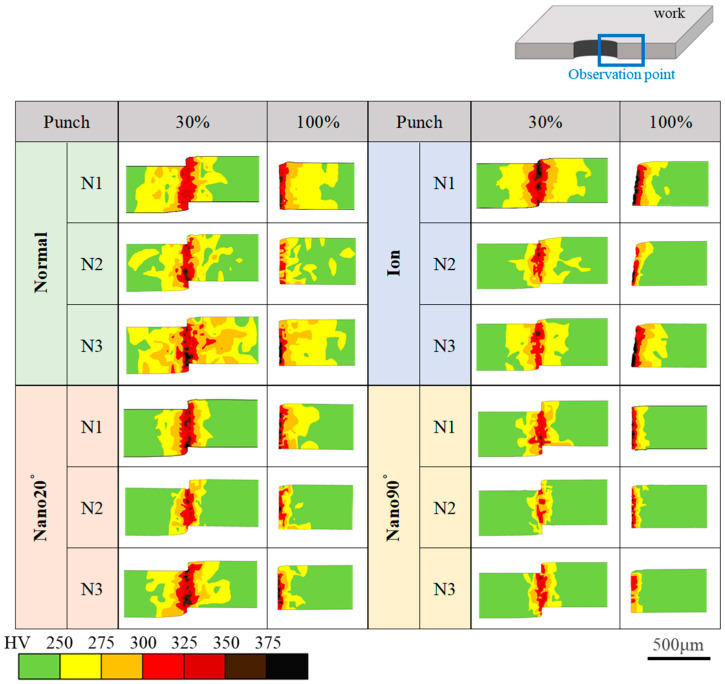
Hardness map of transients of the work hardening zone from a sheared state (30%) to a fully sheared state for the four punching conditions. Three shots—N1, N2, and N3—were performed in each shearing experiment.

A short-shot shearing experiment using 30% of the full stroke was performed to describe the transients of the work hardening process compared to fully punching-out. Three shots (N1, N2, and N3) were made with a constant clearance of CL2.5 μm in Figure 11.

When shearing with the normal punch, the medium work hardening zone fluctuates by itself among the three shots. This fluctuation is also seen on the cross-section after fully punching out. This implies that the metal flow of the work materials is significantly affected by the heterogeneous edge profile of the normal punch.

In the case of the ion punch, the medium work hardening zone is narrowed, with lower fluctuation among the three shots after short-shot when compared to normal punching. This zone of the short-shot specimen is reduced after fully punching out. This is because a part of the work hardening zone has been determined to be elastic in the unloading process after fully punching out. It should be noted that the highest work hardening zone increased after punching out. This implies that the shear strain localizes along the shearing line due to the sharpened edge of the ion punch.

In the case of the Nano20° punch, the medium work hardening zone becomes larger in the N3 test than in the N1 and N2 tests in the short-shot specimen. This zone is much reduced after punching out. This reveals that most of the medium work hardening zone is elastic in the unloading process after fully punching out. In fact, the highest work hardening zone is commonly seen in the vicinity of the sheared surface, even after punching out. This proves that the shearing process using the Nano20° punch affects strain concentration in a similar manner to shearing via the ion punch.

When using the Nano90° punch, the medium work hardening zone area is greatly narrowed; there are not the high work hardening zones even in the vicinity of the sheared surface. In addition, no significant fluctuation of the work hardening zones is detected after the short-shot and fully punching out experiments.

**Figure 12:**
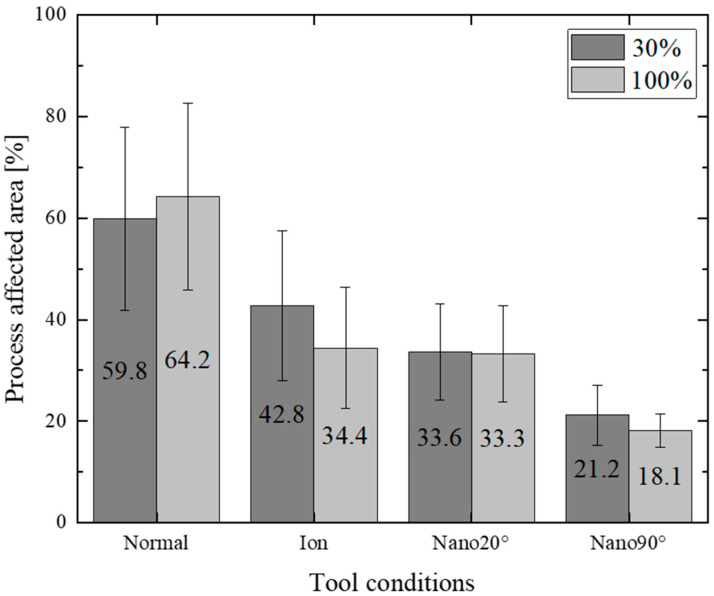
Effect of tool conditions on the work hardening damage fraction at the short-shot after fully punching out.

Figure 12 compares the work hardening zone area fractions after short-shot (30%) and fully punching out (100%) experiments for the four punches.

The normal punch induces the largest work hardening zones due to its heterogeneous edge profile. In the case of the ion punch, the total work hardening zone is reduced by 64.4% to 43.8% after fully punching out due to its homogeneously sharpened edge. When using the Nano90° punch, far fewer work hardening zones are induced to be 20% along the shearing line. This difference in the work hardening process among the four punches comes from the difference in the metal flow pattern during shearing. The plastic work dissipation might well be a measure by which to describe the punch edge profile’s effect on the shearing process.

## 4. Discussion

The tool edge configuration and the clearance have influence on the work hardening damage inflicted on the sheared electrical steel sheets. When using the mechanically ground normal punch, the widest work hardening volume is depicted in Figure 9 and Figure 11, irrespective of the clearance. This is because the work plastic flow around the heterogeneous punch edge enhances the work hardening. Figure 12 also shows the largest deviation in the work hardening zone area fractions for three shots. The heterogeneous punch edge also exaggerates the mechanical fluctuation in the work hardening behavior from experiment to experiment.

When using the ion-sharpened punch, the severe work hardening zone volume reaches its maximum for the four punches in the case of CL2.5 μm. The fracture surface area ratio also reaches the maximum value in the case of CL25 μm. This implies that the work plastic flow localizes at the sharpened punch edge, results in the most severe work hardening state, and induces the onset of fracture. This local work hardening behavior is also observed when using the Nano20° punch. However, the work hardening zone volume is much smaller in cases of greater clearance than 5 μm. The severe work hardening zone volume does not increase via a decrease in the clearance. The fracture surface area ratio is always smaller than that attained using the ion-punch. This reveals that the nano-grooves in the direction of 20° control the local work plastic flow around the sharped edge and reduce the work hardening damage.

When using the Nano90° punch, the local work hardening zone volume reduces significantly, even in case of a clearance under 5 μm. It becomes smaller than that by 69% when using the normal punch, and smaller than that by 51% when using the Nano20° punch. This drastic suppression of work hardening damages comes from the nano-groove structure with a groove orientation change from 20° to 90°.

When using the Nano20° punch, the surface of the workpiece interacts with the nano-periodic structure formed by the punch tip during shearing. When using the Nano90° punch, on the other hand, the workpiece plastically flows without interaction between its surface and the nano-periodic structure. Therefore, a state of local stress concentration continues until shearing is completed, and the work hardening zone area is considered to be narrow and concentrated in the vicinity of the shearing edge.

The plastic dissipation work (W) provides a measurement by which to analyze the tool edge topology effect on the work hardening damage. As listed in Table 2, W, calculated by Equation (3), reduces monotonously by changing the (normal punch) → (ion-sharpened punch) → (Nano20° punch) → (Nano90° punch). The deviation (ΔW) of W among three strokes in the shearing process represents the effect of homogeneity in the punch edge configuration to the local plastic flow. The monotonous decrease in ΔW, with (normal punch) → (Ion) → (Nano20°) → (Nano90°), proves that local plastic flow is sufficiently well controlled to suppress local work hardening using the sharpened and nano-grooved punch edge.

In comparison to the normal punch, the plastic work (W) is halved when using the Nano20° punch and reduced to one-third when using the Nano90° punch. Let us discuss the effect of the nano-grooved punch edge configurations on W through a comparison to the ion-milled punch. When using this punch, the shear strain exceeds a certain level and induces ductile separation at its sharp and homogeneous edge. The nano-grooves work to enhance the strain concentration at the nano-grooved edges and to onset the shear-slipping process of the work materials from the edges. The difference in the shearing and burnishing behavior between the ion-milled and nano-grooved punches comes from the stress concentration, which is determined by the indentation of the nano-grooves to induce the separation of the work materials. The plastic work is minimized by using these nano-grooved punches.

Regarding the shear processing of non-oriented electrical steel sheets using a nano-grooved punch, a case has been reported in the research of Aizawa et al., in which plasma nitriding was applied to the alloy tool steel SKD11 [17]. This study reports that a non-oriented electrical steel sheet with a thickness of 0.15 mm was sheared along the entire burnished surface. However, the mechanism by which shear stress concentrates on nano-grooved structures has not been considered. In the piercing of amorphous electrical steel sheet, five stacked sheet piercings have been attempted by imparting a nano-periodic structure to the sides of a diamond punch at an angle of 60° from the bottom of the punch [19]. As a result, it was reported that the nano-periodic structure has the effect of suppressing the attraction of the work material to the die side. It is thought that even with a brittle amorphous electrical steel sheet with a thin thickness of 0.025 mm and an elongation of only 0.2%, shear stress is concentrated in the nano-periodic structure at the punch tip, thereby reducing the radial effect of the hole. These reports also suggest that the addition of nano-periodic structures to the punch may be a factor in upgrading the sheared surface as it helps to concentrate the shear stress on the punch edge.

The difference in local shearing and burnishing behavior between Nano20° and Nano90° is considered next. In the case of Nano20°, the locally sheared work material starts to flow along the skewed orientation by 20° on the punch side surface and to separate from the nano-grooves. This work material separation from nano-grooves distributes the shear stresses and induces the broadening of the work hardening zones in the radial direction. On the other hand, no work separation occurs from the 90°-nano-grooved edge to preserve the shear stress concentration at the nano-grooved edges against the work materials. 

The plastic dissipation work is minimized by this local control of the material flow around the nano-grooved edges. This indicates that the continuous contact between the nanometer-sized workpiece and the tool is a significant factor underpinning shear stress concentration.

## 5. Conclusions

Four types of shearing punches are employed to experimentally analyze the effect of clearance and punch-edge topological configurations on the work hardening damage by shearing the non-oriented steel sheets. This conclusion is summarized in the following:

(1) When using a nano-grooved punch, the shear strain concentrates at the edge to induce ductile separation at its sharp and homogeneous edge.
(2) The nano-grooves work to enhance the strain concentration at the nano-grooved edges and to onset the shear-slipping of the work materials from the edges.
(3) When using the Nano90° punch, the fractured surface areas, work hardening zone, and plastic work are minimized.
(4) The addition of nano-grooves to the punch provides an engineering means of upgrading the sheared surface by helping to concentrate shear stress on the punch edge.

## Figures and Tables

**Table 1:** Comparison of the elastic zone (H < 250 HV; green-colored zone in Figure 9), the medium work hardening zone (250 HV < H < 300 HV; yellow- and orange-colored zones in Figure 9), and the high work hardening zone (300 HV < H < 375 HV and H < 375 HV; red, dark-red-, brown-, and black-colored zones in Figure 9) for each clearance and punch.

Tool Conditions	Clearance	Elastic Zone	Medium Work Hardening Zone	High Work Hardening Zone
**Normal**	25 μm	37.5 [%]	50.4 [%]	12.1 [%]
	5 μm	50.0 [%]	39.7 [%]	10.3 [%]
	2.5 μm	26.1 [%]	64.3 [%]	9.6 [%]
**Ion**	25 μm	41.8 [%]	49.7 [%]	8.5 [%]
	5 μm	35.2 [%]	54.5 [%]	10.3 [%]
	2.5 μm	60.5 [%]	25.3 [%]	14.2 [%]
**Nano20°**	25 μm	60.5 [%]	28.8 [%]	10.7 [%]
	5 μm	47.1 [%]	46.9 [%]	6.0 [%]
	2.5 μm	53.3 [%]	38.0 [%]	8.7 [%]
**Nano90°**	25 μm	58.5 [%]	33.5 [%]	8.0 [%]
	5 μm	78.3 [%]	15.5 [%]	6.2 [%]
	2.5 μm	77.2 [%]	15.6 [%]	7.2 [%]

**Table 2:** Comparison of the plastic dissipation work (W).

	Normal	Ion	Nano20°	Nano90°
N1	0.040 [J]	0.063 [J]	0.031 [J]	0.027 [J]
N2	0.042 [J]	0.034 [J]	0.019 [J]	0.008 [J]
N3	0.104 [J]	0.034 [J]	0.046 [J]	0.018 [J]
Average	0.062 [J]	0.044 [J]	0.032 [J]	0.018 [J]

## Data Availability

The original contributions presented in the study are included in the article, further inquiries can be directed to the corresponding author.
